# Exploring the Phytochemical Composition and the Bioactive Properties of Malbec and Torrontés Wine Pomaces from the Calchaquíes Valleys (Argentina) for Their Sustainable Exploitation

**DOI:** 10.3390/foods13121795

**Published:** 2024-06-07

**Authors:** Pablo Ezequiel Tapia, Ana Margarida Silva, Cristina Delerue-Matos, Manuela Moreira, Francisca Rodrigues, Romina Torres Carro, María Daniela Santi, María Gabriela Ortega, María Amparo Blázquez, Mario Eduardo Arena, María Rosa Alberto

**Affiliations:** 1 Instituto de Biotecnología Farmacéutica y Alimentaria (INBIOFAL) CONICET–UNT, Avenida N Kirchner 1900, San Miguel de Tucumán CP 4000, Argentina; pablotapia2108@gmail.com (P.E.T.); rominatc87@gmail.com (R.T.C.); maria.alberto@fbqf.unt.edu.ar (M.R.A.); 2 REQUIMTE/LAQV, ISEP, Polytechnic of Porto, Rua Dr. António Bernardino de Almeida 431, 4249-015 Porto, Portugal; ana.silva@graq.isep.ipp.pt (A.M.S.); cmm@isep.ipp.pt (C.D.-M.); manuela.moreira@graq.isep.ipp.pt (M.M.); francisca.rodrigues@graq.isep.ipp.pt (F.R.); 3 Farmacognosia, Instituto Multidisciplinario de Biología Vegetal (IMBIV), CONICET and Departamento de Ciencias Farmacéuticas, Facultad de Ciencias Químicas, Universidad Nacional de Córdoba, Ciudad Universitaria, Córdoba X5000HUA, Argentina; mdaniela0505@gmail.com (M.D.S.); maria.gabriela.ortega@unc.edu.ar (M.G.O.); 4 Departament de Farmacologia, Facultat de Farmàcia i Ciències de l’Alimentació, Universitat de València, Avd. Vicent Andrés Es-tellés s/n, 46100 Burjasot, Valencia, Spain; amparo.blazquez@uv.es; 5 Facultad de Bioquímica, Química y Farmacia, Universidad Nacional de Tucumán (UNT), Ayacucho 471, San Miguel de Tucumán CP 4000, Argentina

**Keywords:** *Vitis vinifera*, antioxidant, cancer cell cytotoxicity, tyrosinase, lipoxygenase, xanthine oxidase

## Abstract

Hydroalcoholic extracts from Malbec and Torrontés wine pomaces (*Vitis vinifera* L.) originating from the high-altitude vineyards of Argentina’s Calchaquí Valleys were characterized. Total phenolics, hydroxycinnamic acids, orthodiphenols, anthocyanins, non-flavonoid phenolics, total flavonoids, flavones/flavonols, flavanones/dihydroflavonols, and tannins were quantified through spectrophotometric methods, with the Malbec extract exhibiting higher concentrations in most of phytochemical groups when compared to Torrontés. HPLC-DAD identified more than 30 phenolic compounds in both extracts. Malbec displayed superior antiradical activity (ABTS cation, nitric oxide, and superoxide anion radicals), reduction power (iron, copper, and phosphomolybdenum), hypochlorite scavenging, and iron chelating ability compared to Torrontés. The cytotoxicity assessments revealed that Torrontés affected the viability of HT29-MTX and Caco-2 colon cancer cells by 70% and 50%, respectively, at the highest tested concentration (1 mg/mL). At the same time, both extracts did not demonstrate acute toxicity in *Artemia salina* or in red blood cell assays at 500 µg/mL. Both extracts inhibited the lipoxygenase enzyme (IC_50_: 154.7 and 784.7 µg/mL for Malbec and Torrontés), with Malbec also reducing the tyrosinase activity (IC_50_: 89.9 µg/mL), and neither inhibited the xanthine oxidase. The substantial phenolic content and diverse biological activities in the Calchaquí Valleys’ pomaces underline their potentialities to be valorized for pharmaceutical, cosmetic, and food industries.

## 1. Introduction

Agro-industries are currently valued not only due to their productive and economic performance in each country, but also for their relationship and actions with the environment. Although most agro-industrial companies generate wastes whose management or final disposal has been highly questioned in recent decades, they seek to obtain benefits by taking advantage of them, achieving pollution reduction, and generating economic profits derived from the added value that may generate [1,2,3]. In recent years, the food industry has embraced green technologies, clean labeling, and the incorporation of natural ingredients to benefit consumers and the industry itself. In the wine industry, grapes are one of the most produced crops worldwide, with an estimated production of more than 78 million tons in 2020 [2]. It is estimated that for every 100 L of wine, about 30 kg of pomace is generated, mainly consisting of skins, pulp, and stems [4,5]. Although grape pomace can be used for animal feed and compost, among other uses, only a small amount is reused, and its disposal poses environmental problems [6]. Leveraging wine pomace for sustainable purposes represents an effective strategy to mitigate environmental pollution and serves as a substitute to minimize carbon emissions across a winery’s manufacturing process. The biological properties of wine pomace residues are of interest to several industries such as food, cosmetic, and pharmaceutical ones [5].

In previous studies, it was observed that the secondary metabolites present in wine pomace could be used as natural additives due to their antioxidant capacity and ability to improve microbial stability and inhibit the growth of pathogenic microorganisms [5]. Grape pomace extracts offer numerous health benefits due to their antioxidant, anti-inflammatory, anticancer, and hypoglycemic properties [2,5]. These benefits are attributed to the rich contents of nutrients and polyphenols, which underscore their potential as novel pharmacological agents for the treatment and prevention of various diseases [7]. These diseases include cancer, metabolic syndrome, neurological disorders, liver and cardiovascular diseases, and other conditions related to oxidative stress [2,5,7]. Grape pomace is a raw material for the production of dietary supplements (powders, tablets, and capsules), providing an auxiliary source of polyphenols that avoids wine consumption [8]. Among polyphenolic, flavonoids (anthocyanins, flavan-3-ols, and flavonols, among others), tannins and non-flavonoids, such as phenolic acids and stilbenes, are mainly responsible for the well-established antioxidant and anti-inflammatory effects. Due to these properties, phenolic compounds can be used in the cosmetic industry [7,9], inhibiting the enzyme tyrosinase, which is over expressed in skin features such as melasma, freckles, and senile lentigines [10].

According to Wani et al. [7], about 70% of phenolic compounds remain in grape pomace after fermentation-maceration. Therefore, its use and valorization through the extraction of phenolic compounds is an attractive strategy that aims to recover compounds while reducing the environmental impact of their byproducts [11]. However, the biological activity of grape pomace extracts depends on the grape variety, geographical origin, climate, vineyard soil conditions, and the winemaking process [2].

Considering the wide range of possible uses for pomace extracts and the recognition that the polyphenolic composition depends on the plant material’s origin and winemaking techniques, it is of special interest to explore the chemical and functional properties of the pomace of each region and cultivar with the aim of finding industrial applications.

In previous studies, white and red wine pomace extracts from the Calchaquí Valleys were found to inhibit the virulence of pathogenic bacteria (*Pseudomonas aeruginosa* and *Staphylococcus aureus*) [12,13]. These extracts demonstrated an inhibition of *P. aeruginosa*’s swarming motility, biofilm production, and metabolic activity in a biofilm environment. The antibiofilm activities showed a positive correlation with the polyphenol content of the extracts [12]. The Calchaquís Valleys’ wine pomaces also exhibited efficacy against *Leishmania amazonensis*, the agent responsible for American tegumentary leishmaniasis. The extracts also displayed significant anticholinesterase activity, suggesting potentialities for the palliative treatment of Alzheimer’s disease [14].

Given the limited information on the phenolic composition and biological properties of Torrontés and Malbec wine pomaces from this geographical area, the present study aims to investigate the polyphenolic composition, antioxidant capacity, cytotoxicity, and enzyme inhibition potential (tyrosinase, lipoxygenase, and xanthine oxidase) of Torrontés and Malbec pomaces from the Calchaquí Valleys.

## 2. Materials and Methods

### 2.1. Chemicals

For HPLC analysis, methanol and formic acid were HPLC graded from Merck (Darmstadt, Germany). The different standards of phenolic compounds, namely the phenolic acids: gallic acid (≥99%), protocatechuic acid (99.63%), neochlorogenic acid (≥98%), caftaric acid (≥97%), chlorogenic acid (>95%), 4-caffeyolquinic acid (≥98%), va-nillic acid (≥97%), caffeic acid (≥98%), syringic acid (≥98%), p-coumaric acid (≥98%), trans-ferulic acid (≥99%), sinapic acid (≥99%), 3,5-di-O-caffeyolquinic acid (≥95%), ellag-ic acid (≥95%), 4,5-di-O-caffeyolquinic acid (≥90%), cinnamic acid (≥99%); flavonoids: (+)-catechin (≥98%), (-)-epicatechin (≥90%), naringin (≥95%), quecetin-3-O-galactoside (≥97%), quercetin-3-O-glucopyranoside (≥99%), rutin hydrate (≥94%), myricetin (≥96%), quercitrin (≥97%), kaempferol-3-O-glucoside (≥95%), kaempferol-3-O-rutinoside (≥98%), isorhamnetin-3-O-glucoside (≥98%), isorhamnetin-3-O-rutinoside (≥99%), naringenin (98%), quercetin (95%), kaempferol (≥98%), apigenin (≥99%), chrysin (≥99%), tiliroside (≥98%); chalcones: phloridzin dehydrate (99%) and phloretin (≥98.5%); and stilbenoids: trans-polydatin (≥98%), trans-epsilon viniferin (≥95%) and resveratrol (≥99%) were purchased from Sigma-Aldrich (Steinhemin, Germany) and Extrasynthese (Genay, Cedex, France). Their stock solutions were prepared in methanol at concentration levels ranging from 1 to 5 g/L and stored at −20 °C.

Dimethylsulfoxide (DMSO), Triton X-100, 2,2′-Azinobis-(3-ethylbenzothiazoline-6-sulfonic acid) cation radical (ABTS˙+), Trolox (6-hydroxy-2,5,7,8-tetramethylchroman-2-carboxylic acid), Butylated hydroxytoluene (BHT), EDTA, Folin–Ciocalteau reagent, Tyrosinase from mushroom, kojic acid, L-tyrosine, xanthine oxidase from bovine milk, xanthine, nitroblue tetrazolium chloride (NBT), phenazine methosulphate (PMS), β-nicotinamide adenine dinucleotide (NADH), dihydrorhodamine 123 (DHR), and 3-(4,5-dimethylthiazol-2-yl)-2,5-diphenyltetrazolium bromide (MTT) were purchased from Sigma-Aldrich (St. Louis, MO, USA). Other chemicals were obtained from local commercial sources, and were of analytical-grade quality.

Caco-2 cells (clone type C2Bbe1) were acquired from the American Type Culture Collection (ATCC, Manassas, VA, USA), and HT29-MTX was offered from Dr. T. Lesuffleur (INSER-MU178, Villejuif, France). Dulbecco’s Modified Eagle Medium (DMEM), Fetal Bovine Serum (FBS), Hank’s Balanced Salt Solution (HBSS), non-essential amino acids, penicillin, streptomycin, and trypsin–EDTA were obtained from the Invitrogen Corporation (Life Technologies, S.A., Madrid, Spain).

### 2.2. Material Collection

Samples of Malbec and Torrontés grape pomaces were gathered from Albarossa Winery, situated in Tafí del Valle, Tucumán, Argentina, during the 2019 and 2020 harvests. Notably, the vineyard cultivation practices at this winery refrain from the use of chemical substances. The grapes exhibited robust health, with no signs of *Botrytis cinerea* detected. The assessment for the presence of *B. cinerea* was conducted visually, and its absence can be attributed to the windy and arid conditions characteristic of the Calchaquí Valleys region.

The Calchaquí Valleys, located in the northwest region of Argentina, have a temperate climate with notable thermal amplitudes and, occasionally, experience prolonged late frosts in spring. Their high-altitude vineyards range from 1700 m to 2400 m. The grapes were grown on soils characterized as sandy loam or sandy with a high proportion of fine sand. The soil profile is deep, with a somewhat rocky subsoil ensuring excellent permeability and the leaching of salts [15].

The regional white wines correspond to the Torrontés varietal (*Vitis vinifera* L.), which has become the emblematic variety of the region. This grape is optimal to produce aromatic wines and adapts very well to the entire area, becoming the most cultivated one in the region [16]. On the other hand, the Malbec wine varietal (Argentina’s most exploited purple grape variety) from the Calchaquí Valleys shows different particularities from other country wine regions due to the terroir characteristics described above.

Notably, Torrontés pomace was obtained from a white wine-making process, meaning that grapes were not subjected to ethanolic fermentation. In contrast, Malbec pomace was acquired from a red wine-making process, where grapes are entirely involved in fermentation. The collected specimens were stored at −80 °C before being oven-dried at temperatures below 45 °C for 48 h, ensuring the preservation of phenolic compounds until a constant weight was attained. Moisture was calculated using the weight difference before and after drying. The dried material was crushed with a grinder until reaching a particle size < 250 µm (sieve mesh 60). The extract was prepared from this pomace powder.

### 2.3. Preparation of Extracts

The active principles were extracted using hydroalcoholic maceration (ethanol:water, 50:50 *v*/*v*) in a solid–liquid ratio of 1/4, *w*/*v*. Two successive extractions were performed by shaking at room temperature (150 rpm/min) for 3 h. The extracts were vacuum-filtered using Whatman No. 4 filters and evaporated to dryness through vacuum evaporation and subsequent lyophilization. The extracts obtained during the processes were stored in the dark at 4 °C until use. The extraction yields were expressed in mg of soluble principle per gram of dry pomace (mg/g DP) and were calculated as follows: the weight of extract obtained (mg)/the initial weight of the plant matter to be extracted (mg).

### 2.4. Phytochemical Analysis

#### 2.4.1. Quantification of Different Phenolic Groups

For this assay, stock solutions of 2.5 mg/mL of each extract were used. Total extractable phenols and nonflavonoid compounds were determined colorimetrically using Folin–Ciocalteu’s reagent at 765 nm [17]. A standard curve was performed with gallic acid (2–20 µg/mL) as the standard, and the results were expressed in mg of gallic acid equivalents (GAE) per g of dry pomace extract (DPE) (mg GAE/g DPE) and g of dry pomace (mg GAE/g DP) (R^2^ = 0.9965, *p* ≤ 0.05). To eliminate the potential sugar interference, the Torrontés extract was purified using solid-phase extraction prior to Folin–Ciocalteu’s analysis. The C18 cartridge (Waters’ Sep-Pak Cartridges) was activated with 2 mL of methanol and then 5 mL of deionized water. Then, 9 mL of the extract (2.5 mg/mL) was injected and eluted using 9 mL of acidic deionized water (0.1% formic acid). Finally, 9 mL of acidic methanol (0.1% formic acid) was used to recover the polyphenol compounds [18].

The total flavonoid content was determined using sodium nitrite (5%) and aluminum chloride (10%). The technique is based on the formation of specific colored complexes between the flavonoids and the reagents (NaNO_2_ and AlCl_3_), with the color intensity measured spectrophotometrically at 510 nm [19]. For quantification, a quercetin standard curve (4–80 µg/mL) was used and the results were expressed as mg of quercetin equivalents (QE) per g of extract (mg QE/g DPE) (R^2^ = 0.9939, *p* ≤ 0.05)

The flavone and flavonol contents were evaluated spectrophotometrically at 425 nm with aluminum chloride (5%) [17,20]. This technique is based on the formation of a complex between the aluminum ion, Al (III), and the carbonyl and hydroxyl groups of the flavonoid. The standard curve was performed with quercetin (5–40 µg/mL), and the results were expressed in mg of quercetin equivalents (QE) per g of extract (mg QE/g DPE) (R^2^ = 0.9941, *p* ≤ 0.05).

The flavanone and dihydroflavonol contents were measured at 495 nm [17,20]. This technique is based on the reaction of these compounds with 2,4-dinitrophenylhydrazine (DNP) in an acidic medium to form colored phenylhydrazones. The standard curve was performed with naringenin (20–200 µg/mL), and the results were expressed in mg of naringenin equivalents (NE) per g of extract (mg NE/g DPE) (R^2^ = 0.9998, *p* ≤ 0.05).

Orthodiphenols were assessed by UV-Visible spectrophotometry using the sodium molybdate method at a wavelength of 370 nm [21]. A standard curve was performed with caffeic acid (2–20 µg/mL), and the results were expressed as mg of caffeic acid equivalents (CAE) per g of extract (mg CAE/g DPE) (R^2^ = 0.9981, *p* ≤ 0.05).

Hydroxycinnamic derivates were evaluated at 320 nm using caffeic acid (0.5–5 µg/mL) as the standard, and the results were expressed as mg of caffeic acid equivalents (CAE) per g of extract (mg CAE/g DPE) (R^2^ = 0.9972, *p* ≤ 0.05) [22].

The assessment of the total anthocyanin content was carried out using the pH differential method, and the results were expressed as mg of cyanidin-3-glucoside equivalents per g of extract (mg C3GLE/g DPE) according to Carullo et al. [23]. Results were determined by means of the following formula:

(1) $$mg\;C3GLE/L\;DPE=\left[\frac{A\;\times \;MW\;\times \;DF\;\times \;1000}{\varepsilon \;\times \;1\;}\right]$$

where:

A = (A520 nm − A700 nm)pH = 1 − (A520 nm − A700 nm)pH = 4.5; MW (molecular weights of cyanidin-3-glucoside) = 449.2 g/mol; DF = dilution factor; *ε* = (molar extinction coefficient) = 26,900 L/mol cm and 1000 = conversion factor from g to mg.

The determination of the tannin content of the extracts was performed as reported previously by Bouabid et al. [24] using the vanillin assay. A standard curve was performed with catechin (5–25 µg/mL), and the results were expressed as mg of catechin equivalents per g of extract (mg CE/g DPE).

#### 2.4.2. Identification of Phenolic Compounds through HPLC-DAD Analysis

The HPLC analyses were carried out in a Shimadzu HPLC system (Shimadzu Corporation, Kyoto, Japan) equipped with a LC-20AD prominence pump, a DGU-20AS prominence degasser, a CTO-10AS VP column oven, a SIL-20A HT prominence autosampler, and an SPD-M20A photodiode array detector.

The phenolic profile of the obtained extracts was analyzed according to the method described by Moreira et al. [25] with slight modifications. Chromatographic analyses were performed using a Shimadzu HPLC system, and polyphenol separation was achieved on a Gemini C18 column (250 × 4.6 mm, 5 μm) from Phenomenex at 25 °C. The solvent system used, pumped at a flow rate of 1 mL/min, was methanol (eluent A) and water (eluent B) with both acidulated with formic acid (0.1%), and the following gradient was employed: 0–5 min: 20–24% A; 5–7 min: 24–25% A; 7–10 min: 25–26% A; 10–11 min: 26–26.5% A; 11–18 min: 26.5% A; 18–25 min: 26.5–30% A; 25–50 min: 30–45% A; 50–60 min: 45–50% A; 60–70 min: 50–55% A; 70–90 min: 55–70% A; 90–100 min: 70–100% A, followed by 100% A for 5 min, back to 20% A in 10 min and 5 min of reconditioning before the next injection. Individual phenolic compounds were identified by comparing the samples’ retention time and UV-Vis spectra with those from pure standards. Chromatograms were recorded at 280, 320, and 360 nm depending on the maximum absorption of the phenolic compound identified. Before injection, the dried extract was resuspended in methanol/water (20:80) and filtered through a 0.22 μm PTFE syringe filter. The quantification of phenolic compounds was made based on calibration curves of the pure standards, and results were expressed as mg of compound per 100 g of DPE.

### 2.5. Antioxidant Capacity Assays

At least three independent experiments were performed for each method. For the samples and positive controls, six concentrations were analyzed in duplicate. Prior to the assays, the absorption of the extract was studied at the proper wavelengths. GraphPad Prism 7 software (GraphPad, La Jolla, CA, USA) was used to calculate the results based on the curves of the inhibition percentage versus the antioxidant concentration. For chelating, scavenging, and reducing capacities, the concentration of the extracts necessary to chelate, scavenge, or reduce 50% of the radicals or iron ions (IC_50_) was determined through linear regression analysis. If the IC_50_ was not reached, the results were expressed as the percentage of inhibition at the highest concentration tested.

#### 2.5.1. Phosphomolybdenum-Reducing Capacity

The phosphomolybdenum method described by Carullo et al. was used [23]. The absorbance of the green-colored complex was measured spectrophotometrically at 695 nm. The standard curve was performed with ascorbic acid (5–50 µg/mL) as the standard, and the results were expressed in μg of ascorbic acid equivalents (AAE) per mg of DPE (μg AAE/mg).

#### 2.5.2. Metal-Chelating Capacity

The metal chelating capacity is based on the formation of colored Fe^2+^ complexes whose concentrations can be determined spectrophotometrically. Molecules present in a sample that can chelate iron will compete with ferrozine, decreasing the reaction’s coloration (absorbance) [17]. The absorbance was measured at 562 nm. EDTA (5–20 μg/mL) was the positive control used.

#### 2.5.3. ABTS Cation Radical-Scavenging Capacity

The assay to determine the ability of the extracts to scavenge the 2,2′-Azinobis-(3-ethylbenzothiazoline-6-sulfonic acid) cation radical (ABTS˙+) was carried out according to a method previously described [17]. The absorbance was measured at 750 nm, and the percent purification was calculated at 6 min. Trolox (2–7 μg/mL) was used as a positive control.

#### 2.5.4. Nitric Oxide-Scavenging Capacity

The method described by Torres-Carro et al. [17] was used to determine the nitric oxide depurating capacity of the extracts. This technique uses a Griess reagent to give a pink-colored azo complex with a maximum absorption at 550 nm. Ascorbic acid (25–200 μg/mL) was used as a positive control.

#### 2.5.5. Iron-Reducing Power

The ability of the residue extracts to reduce potassium ferricyanide (Fe^3+^) to potassium ferrocyanide (Fe^2+^), which forms a Prussian blue complex, was detected spectrophotometrically at 700 nm. The absorbance values were used to determine the concentration required to reduce 50% of the Fe^3+^ (RC_50_). BHT (3–13 μg/mL) was used as a positive control [17].

#### 2.5.6. Copper-Reducing Power

The CUPRAC assay was performed utilizing the copper(II)-neocuproine (Cu(II)-Nc) reagent as the chromogenic oxidant [26]. The standard curve was performed with gallic acid (0.5–5 μg/mL), and the results were expressed in μg of gallic acid equivalents (GAE) per mg of DPE (μg GAE/mg).

#### 2.5.7. Superoxide Anion Radical-Scavenging Assay

The quenching ability of superoxide anion radical (O_2_^●−^) was assessed based on the NBT reduction into purple-colored diformazan [27]. O_2_^●−^ was produced by the non-enzymatic NADH/PMS/O2 system. The absorbance was measured at 560 nm at 37 °C for 5 min. Results were expressed as the inhibition, in IC_50_, of the NBT reduction to diformazan. As positive controls, catechin (10–100 μg/mL) and gallic acid (10–100 μg/mL) were employed.

#### 2.5.8. Hypochlorous Acid-Scavenging Assay

The quenching abilities of the samples and the positive controls against hypochlorous acid (HOCl) were determined through a procedure previously described [27]. DHR, used as a fluorescence probe, was oxidized to rhodamine by HOCl. A 1% (*m*/*v*) NaOCl solution was used after adjusting the pH to 6.2. The inhibition of the HOCl-induced oxidation of DHR was determined. Catechin (0.05–0.5 μg/mL) and gallic acid (0.5–2.0 μg/mL) were employed as positive controls.

#### 2.5.9. Saccharomyces Cerevisiae Survival Assay

Yeast cells were exposed to oxidative stress induced by 2 mM of H_2_O_2_ in the presence and absence of an extract. Two controls were used: yeast exposed to the vehicle of the extract (DMSO) and yeast exposed to extracts without the addition of H_2_O_2_. Cell viability was analyzed by determining the CFU/mL in a solid medium [28]. The results are expressed as a percentage of survival. One-hundred-percent survival is defined as the CFU/mL observed on the control plate, which contains yeast exposed to an extract vehicle, without extracts or hydrogen peroxide.

### 2.6. Toxicity Trials

#### 2.6.1. Artemia Salina Test

The acute toxicity levels of Torrontés and Malbec pomace extracts, with concentrations from 250 to 500 μg/mL, were evaluated using the brine shrimp lethality test [17]. The experiments for each concentration were conducted in triplicate. The negative control wells contained DMSO to a final concentration lower than 0.3%, and the positive control potassium dichromate (10–40 μg/mL). Survival percentages were calculated by comparing the number of survivors in the test wells with respect to the negative control.

#### 2.6.2. Hemolysis

The hemolytic impact of the pomace extracts was assessed spectrophotometrically at 550 nm following the method described by Torres-Carro et al. [17]. Extracts ranging from 200 to 1000 μg DPE/mL, or the vehicle (serving as a 0% hemolysis control), were brought into contact with a 10% suspension of human red blood cells (HRBC). A number of 100% hemolysis controls were established by exposing the 10% HRBC suspension to deionized water and to 1% (*w*/*v*) Triton X-100.

#### 2.6.3. Cell Viability Assay

The 3-(4,5-dimethylthiazol-2-yl)-2,5-diphenyltetrazolium bromide (MTT) assay was performed to evaluate the effect of the different extract concentrations (0.1–1000 µg DPE/mL) on the intestinal cell lines. Passages 69–71 and 31–32 were used for Caco-2 and HT29-MTX, respectively. Briefly, cells (25 × 10^3^ cells/mL) were incubated for 24 h with fresh medium in the absence or presence of the extracts dissolved in a cell culture medium.

Following the extracts’ removal from each well, cells were washed with HBSS. The number of viable cells was determined by adding MTT reagent and incubating for 3 h at 37 °C. DMSO was used to solubilize the crystals. The positive control was DMEM, and the negative control was 1% (*w*/*v*) Triton X-100. Cells were grown according to the methodology described by Pinto et al. [27]. The absorbance was read at 590 nm with background subtraction at 630 nm. Results were expressed as percentages of cell viability.

### 2.7. Enzyme Inhibitions

The enzyme inhibition was calculated as a percent as follows: % inhibition = [(Abs control − Abs sample)/Abs control] × 100, where Abs control is the absorbance of the control solution and Abs sample is the absorbance of the sample solution. When possible, the concentration responsible for inhibiting 50% of enzyme activity (IC_50_) was established through regression analysis, employing a concentration–inhibition response curve.

#### 2.7.1. Tyrosinase

The tyrosinase inhibitory capacity of Torrontés and Malbec pomace extracts was performed as previously described by Matos et al. [6]. The absorbance was measured at 475 nm after 20 min of incubation. Kojic acid (0.1–5 µg/mL) was used as a positive control agent.

#### 2.7.2. Xanthine Oxidase

The assay was conducted as previously described Quy and Xuan [29]. The absorbance was measured at 290 nm in a spectrophotometer. The reference inhibitor Allopurinol (0.05–5 µg/mL) was used as a positive control.

#### 2.7.3. Lipoxygenase

The assessment of LOX activity followed the methodology outlined by Torres-Carro et al. [17]. Soybean LOX was exposed to different concentrations of the extracts or the vehicle and its substrate, linoleic acid. The inhibitory potential was gauged by computing the percentage of hydroperoxide production inhibition at 234 nm. Quercetin (40–70 µg/mL) and gallic acid (30–80 µg/mL) were used as positive controls.

### 2.8. Statistical Analysis

All data were expressed as the mean ± standard deviation from at least three independent experiments. The HPLC statistical analysis was conducted using IBM SPSS Statistics 26.0 software (SPSS Inc., Chicago, IL, USA). For the other assays, the statistical analysis was performed using INFOSTAT Analytical Software version 2020e (Universidad Nacional de Córdoba, Córdoba, Argentina). Differences in mean values were evaluated using Student’s *t*-test for independent samples. In all analyses, *p*-values < 0.05 were considered statistically significant.

## 3. Results and Discussion

### 3.1. Chemical Composition

*Vitis vinifera* byproducts are an important source of phytochemicals with potential health-promoting properties and biotechnological interests. The chemical compositions and health benefits of several grape pomaces have been previously reported [5]. However, little is known about the phytochemical profiles of Malbec and Torrontés wine pomaces from the Calchaquí Valleys.

In the present study, the Malbec and Torrontés pomace samples had moistures between 65 and 70%, and the oven-drying over freeze-drying was selected due to its potential applicability within the wine industry and cost-effectiveness [30]. After drying and milling into flour, the grape pomaces were extracted with the use of non-toxic solvents (ethanol/water) and dried. Green solvents were used to acquire and characterize polyphenolic extracts suitable for human consumption or utilization in medical, cosmetic, or pharmaceutical sectors. Additionally, it is noteworthy that ethanol is a byproduct of the wine industry. The extraction yields of soluble compounds from the Malbec and Torrontés pomaces were 161.6 ± 16.7 and 735.2 ± 51.2 mg of extract/g of flour, respectively (16% and 73%). The extraction yields, and the total phenolic contents align with the results obtained using the same solvent (ethanol 50%) for other red wine pomaces, with values ranging between 5.3% and 16.1% [31,32]. Furthermore, the Torrontés pomace yields were significantly higher than those reported for other white wine pomaces, which ranged between 4.9% and 7.4% [32], having probably a higher content of extractable compounds than polyphenols. It is worth noting that this yields discrepancy may be attributed to the fact that, in the cited study, the authors chose to wash the pomace with water before drying to eliminate residual sugar, a step that was not carried out in the present work.

The analysis of the phenolic metabolites identified in the pomace extracts is detailed in Table 1. A comparison between the two analyzed pomace varieties reveals that the red Malbec variety exhibited a total phenol content 6.5 times higher than that of the Torrontés. Non-flavonoid phenolic compounds accounted for 19.5% of the total polyphenol content in the Malbec pomace extract, while in the Torrontés, these compounds represented 40.2%. The total flavonoid compound content in the Malbec extract surpassed that of the Torrontés 7.5 times. Interestingly, the tannin content was almost similar in both varieties. It should be noted that no anthocyanins were detected in the Torrontés extract (<LOQ).

The concentration of total polyphenols in the Malbec dry extract (156.01 mg GAE/g DPE) was very similar to the findings in a Malbec pomace from Mendoza, Argentina (the Cuyo region), which reported 196.2 mg of GAE/g of DPE) [31]. The present findings align with the total polyphenol levels (ranging between 127 and 298 mg/g of DPE) and the total flavonoid content (ranging between 137 and 322 mg/g of DPE) observed in four other red skin pomaces derived from Italian cultivars (Barbera, Grignolino, Pinot Noir, and Nebbiolo). Nevertheless, in the present study, the tannin content was lower [32]. Furthermore, the results achieved exceed those reported for Merlot pomace from Brazil [33] in terms of total polyphenols, total flavonoids, and anthocyanins. It is worth noting that all these studies utilized extraction conditions like the present research. Higher values of total polyphenols (523 mg/g DPE), but lower levels of anthocyanins (1.67 mg/g DPE), were reported for Cabernet Sauvignon grape pomace (Mexico) that was defatted with hexane prior to extraction with ethanol at 60% [30].

Likewise, Torrontés pomace from Galicia (extracted with 65% methanol) exhibited a lower concentration of total phenolic compounds (22 mg GAE/g DPE) as reported by Alvarez-Casas et al. [34], which is consistent with the present findings (19.9 mg GAE/g DPE). The sugars present in phenolic-extracted Torrontés grape marc lead to a reduced polyphenol richness per mass of extract when compared to the red pomace extract. Guaita et al. [32] documented higher values of total phenolics (144–208 mg/g DPE), total flavonoids (108–206 mg/g DPE), and tannins (64–108 mg/g DPE) for three other white-skinned pomaces of Italy (Muscat blanc, Arneis, Cortese) extracted with 50% ethanol. Variations in extraction methods, grape types, and agroclimatic conditions contribute to disparities in the measured total polyphenols, explaining the divergent values reported in the literature. Moreover, the genotype is the main factor that influences the relative concentrations of the different phenolic compounds [35].

Through HPLC-DAD analysis, 35 individual phenolic compounds were identified in the extracts, including 14 phenolic acids, 18 flavonoids, and 3 stilbenoids (Table 2). Most of the compounds identified were found in both pomaces. In the Torrontés extract, the main polyphenols were 4,5-di-O-caffeoylquinic acid ˃ 4-O-caffeyolquinic acid ˃ kaempferol-3-O-glucoside ˃ isorhamnetin-3-O-glucoside ˃ caftaric acid ˃ sinapic acid ˃ gallic acid ˃ ferulic acid ˃ protocatechuic acid ˃ (+)-catechin. Regarding the Malbec extract, the main phenolic compounds were protocatechuic acid ˃ (+)-catechin ˃ gallic acid ˃ 4-O-caffeyolquinic acid ˃ sinapic acid ˃ 4,5-di-O-caffeoylquinic acid.

The phenolic patterns among the varieties exhibited notable quantitative differences. For instance, in the Torrontés extract, the content of 4,5-di-O-caffeoylquinic acid is five times higher compared than that of the Malbec extract. Conversely, the Malbec extract displayed significantly elevated levels of (+)-catechin, protocatechuic acid, and gallic acid, which were 8.8, 9.8, and 2.7 times higher, respectively, than those found in the Torrontés extract. Additionally, in the pomace of another red grape variety, Cabernet Sauvignon, quercetin, catechin, epicatechin, and syringic acid emerged as dominant phenolic compounds [36].

To contextualize our findings against other published data for red and white whole pomaces (skin and seeds) extracted with hydroalcoholic solvents, the results were presented in the milligrams of the compound per 100 gram of dry pomace extract (mg/ 100 g DPE) or per 100 gram of dry pomace (mg/100 g DP) (Table 2). The results attested that gallic acid (2.74–73 mg/g DPE and 6.4–14.07 mg/g DP), protocatechuic acid (≤0.29 mg/g DPE and 0.6–2.03 mg/g DP), chlorogenic acid (≤0.06 mg/g DPE and ≤0.13–0.23 mg/g DP), and ferulic acid (2.4 mg/g DPE and <0.13 mg/g DP) are present in concentrations much higher than the values reported for red wine pomaces [14,30,31,33,37,38,39]. Furthermore, the phenolic acids: gallic (3.3–11.12 mg/g DP), protocatechuic (0.39–7 mg/g DP), caffeic (falling within the range of 0.15 mg/g DP), caftaric (1.9–7.9 mg/g DP), and ferulic (0.13–0.22 mg/g DP); and the flavonoids quercetin-3-O-galactoside (approx. 0.96 mg/g DP) and kaempferol-3-O-glucoside (approx. 0.21 mg/g DP) are found in much higher concentrations than those reported in the literature for white wine pomaces [14,34,39]. Although resveratrol has not been identified, the presence of derivatives such as polydatin, also known as piceid (a resveratrol derivative with improved bioavailability), and trans-epsilon viniferin (a resveratrol dimer) has been detected. To the best of our knowledge, this study reports for the first time the presence of neochlorogenic acid, 4-O-caffeoylquinic acid, 3,5-di-O-caffeoylquinic acid, 4,5-di-O-caffeoylquinic acid, sinapic acid, and isorhamnetin-3-O-rutinoside in hydroalcoholic extracts of wine pomace.

Malbec wine pomace from Argentina’s Cuyo region exhibited lower levels of catechin (338 mg/100 g DPE) and gallic acid (25 mg/100 g DPE) compared to the present findings for the Malbec pomace from the Calchaquí Valleys. In contrast, Cuyo’s Malbec wine pomace displayed higher concentrations of epicatechin (176 mg/100 g DPE) and syringic acid (173 mg/100 g DPE). It is interesting to note that protocatechuic acid, the main phenolis acid observed in this study, was not reported in the Malbec pomace from Cuyo [31]. In another study [14] that assessed the polyphenol content in a concentrated chromatographic fraction obtained from a methanolic extract of red pomace from the Calchaquí Valleys (of an unknown variety), C6-C1 phenolic acids, gallic acid, and syringic acid were identified as the main phenolics. In agreement with the present results, Teixeira et al. [35] reported protocatechuic acid as the most abundant hydroxybenzoic acid in pomace from red varieties.

Regarding white wine pomace from the Calchaquí Valleys (of an unknown variety), Salazar et al. [14] highlighted gallic acid as the predominant phenolic acid. In contrast, the results obtained in this work reveal a distinct composition, with an emphasis on the prevalence of derivatives of caffeoylquinic acid, contrasting with Salazar’s observations. In Galician Torrontés pomace, phenolic acids such as gallic, protocatechuic, and caftaric acids, and the flavonoids epicatechin, quercetin, and quercetin derivatives were identified [34]. In the present work, a greater diversity of phenolic compounds was explored, which explains why other authors do not report the caffeoylquinic acid-derived compounds which quantitatively were some of the most important in the present research.

A literature review [35] described notable distinctions in the hydroxycinnamic acid content between red and white grape skins. Specifically, white grape skins exhibited elevated levels of cis-coutaric acid and trans-caftaric acid, whereas red grape skins were predominantly composed of chlorogenic acid (3-O-caffeoylquinic acid). This study observed similar chlorogenic acid contents in both varietals, while caftaric acid was only present in the Torrontés pomace.

### 3.2. Biological Activity

#### 3.2.1. Antioxidant

**Figure 1:**
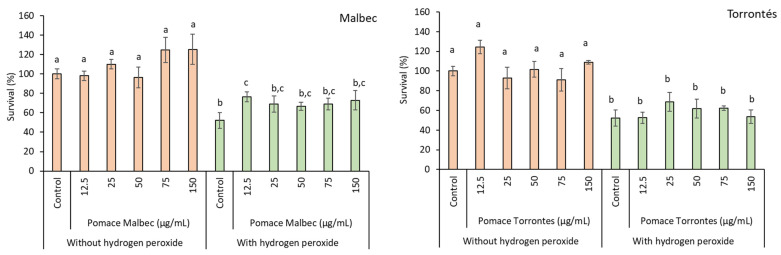
*Saccharomyces cerevisiae* survival in the presence of different pomace extract concentrations (from 12.5 to 150 µg DPE/mL). Different letters indicate significant differences between treatments (*p* < 0.05), according to Tukey’s test.

Reactive species, particularly reactive oxygen species (ROS) and reactive nitrogen species (RNS), play a significant role in various physiological processes, including cell signaling, inflammatory cascades, and homeostasis. Consequently, the evaluation of the scavenging capacity of an extract against ROS and RNS becomes more intriguing due to their pivotal functions in living tissues. The antioxidant and antiradical activities of hydroalcoholic extracts were evaluated in nine different assays (Table 3 and Figure 1). In the ABTS cation radical, nitric oxide, superoxide anion, and hypochlorite assays, the Malbec extract demonstrated the best scavenging efficiency, as indicated by its lower IC_50_ values. In the iron-chelating assay, the Malbec extract could chelate 42% of the metal at 1000 µg/mL (Table 3). Additionally, the Malbec extract exhibited superior Fe^3+^- and Cu^2+^-reducing power when compared to the Torrontés extract (Table 3). To assess the bioactivity of wine pomace in safeguarding Saccharomyces cerevisiae cells from induced oxidative damage, cell viability was measured in the presence or absence of extracts such as chemoprotectors. As depicted in Figure 1, the selected concentrations from the samples (12.5–150 µg/mL) exhibited non-cytotoxicity to *S. cerevisiae*. Upon the induction of oxidative stress, yeast cells demonstrated sensitivity to H_2_O_2_, with only 50% surviving the oxidative insult. Malbec grape pomace extract (12.5 µg/mL) rescues 24% of yeast from oxidative stress induced by H_2_O_2_, while the Torrontés pomace did not show a protective effect.

In summary, the Malbec grape pomace demonstrated significant scavenging activity against ROS and RNS, potentially attributed to its higher phenolic content when compared to the Torrontés grape pomace, particularly with concerns to catechin, epicatechin, gallic acid, and protocatechuic acid whose free radical-scavenging potential has been extensively demonstrated [2]. This distinction could account for the heightened antioxidant activity observed in this particular varietal.

The divergence in the antioxidant and antiradical activities between red and white pomace extracts is attributed to their distinct phenolic profiles, significantly influenced by the grape variety and extraction methods [40]. The free radical-scavenging activity and the ferric-reducing power of grape pomace are correlated with specific phenolic acids (gallic and caffeic), flavan-3-ols (catechin, epicatechin, and galloylcatechin), and flavonols (quercetin) [18,38].

The antioxidant activity results obtained in this study surpassed those reported by other authors for hydroalcoholic extracts of red wine pomace (Syrah, Petit Verdot, and Romy) and white wine pomace (Chenin Blanc and Banaty): ABTS scavenging (IC_50_ 56.22 µg/mL for Romy and 78.47 µg/mL for Banaty), iron-reducing ability (RC_50_ 160.97 µg/mL for Romy and 141.5 µg/mL for Banaty), iron-chelating capacity (CC_50_ 262.67 µg/mL for Romy and 248.35 µg/mL for Banaty), superoxide anion scavenging (IC_50_ 190 µg/mL for Petit Verdot, 240 µg/mL for Syrah, and 2160 µg/mL for Chenin Blanc), and hypochlorite scavenging (IC_50_ 17 µg/mL for Petit Verdot, 31 µg/mL for Syrah, and 128 µg/mL for Chenin Blanc) [41,42]. No reports were found regarding nitric oxide-scavenging capacity. Pomace extracts from Syrah, Merlot, and Cabernet Sauvignon boosted the *S. cerevisiae* survival rate by 8% to 16% compared to cells exposed to H_2_O_2_ [28].

Protocatechuic acid, catechin, gallic acid, ferulic acid, and chlorogenic acid, present in the Calchaquíes Valleys’ wine pomaces, exhibit various pharmacological activities. These include antioxidant, anti-inflammatory, neuroprotective, antibacterial, antiviral, anticancer, antiosteoporotic, analgesic, antiaging, antihypertensive, anti-diabetic, antihyperlipidemic, anticoagulant, antiulcer, cardioprotective, and hepatoprotective properties [43,44,45,46,47,48]. The antioxidant properties of the pomace extracts observed, particularly those of the Malbec, are partially attributed to the high content of these phenolic compounds.

Additionally, accumulated evidence demonstrated that caffeoylquinic acids present in high concentrations in the Torrontés pomace extract have a wide range of biological activities, such as antiparasitic, antioxidation, antiviral, antibacterial, anti-inflammatory, anticancer, neuroprotective, and anti-diabetic effects [48]. Di-O-caffeoylquinic acids protect bone marrow-derived mesenchymal stem cells from •OH-induced damage and the antioxidant mechanisms include electron transfer, H^+^ transfer, and Fe^2+^ chelation [49,50].

Likewise, polydatin has demonstrated countless pharmacological properties, primarily including anticancer, cardioprotective, anti-diabetic, gastroprotective, hepatoprotective, neuroprotective, and antimicrobial effects, along with health-promoting roles in the renal system, the respiratory system, rheumatoid diseases, the skeletal system, and women’s health. This resveratrol derivative has higher antioxidant and anti-inflammatory activity than resveratrol [51].

The proven antioxidant capacity of the hydroalcoholic extracts derived from Malbec and Torrontés pomaces, coupled with their demonstrated antivirulence activity against pathogenic bacteria (*P. aeruginosa* and *S. aureus*) as indicated by other authors [12,13], implies a promising prospect for their use as natural preservatives in the food industry. This potential application could play a crucial role in mitigating oxidative processes.

This collective evidence elucidates the biological activity of the Calchaquíes Valleys’ wine pomaces, underscoring the substantial health-promoting benefits associated with the presence of these diverse and potent phenolic compounds.

#### 3.2.2. Cytotoxicity

The cytotoxic assay performed on HT29 and Caco-2 colon cancer cells is shown in Table 4. The Torrontés extract significantly reduced cell viability in a dose-dependent manner, achieving 67% and 48% viability for HT29 and Caco-2 cells, respectively, at the highest tested concentration (1 mg/mL). In contrast, the Malbec pomace at the same concentration reduced the viability of Caco-2 cells by 20%. These findings align with previous research indicating that grape pomace can inhibit the proliferation of colon adenocarcinoma cells (Caco-2, HT-29) in a dose-dependent manner, and that white grape pomace is more active than red [52].

The anti-proliferative effects of Zalema (white grape) pomace hydroalcoholic extract (methanol 75%), as well as specific phenolic standards like catechin, epicatechin, quercetin, and gallic acid on Caco-2 cells, have been previously documented [53]. Additionally, the anti-proliferative impact of caffeoylquinic acids on human colon cancer cells has been established [49,54]. The observed effects in this study could be attributed, at least in part, to the high content of these acids in the Torrontés pomace. On the other hand, the synergistic effect of different polyphenolic compounds as chemopreventive agents is well-documented [53].

This study represents the first report on the cytotoxic evaluation of pomaces from the Calchaquí Valleys on human colon carcinoma cells and the first description of the anti-proliferative capacity of Torrontés wine pomace.

#### 3.2.3. Toxicity

**Figure 2:**
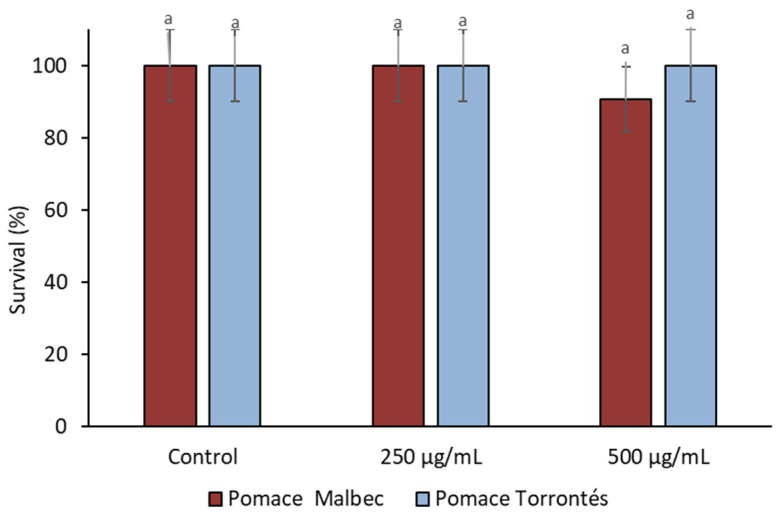
*Artemia salina* survival in the absence and presence of different pomace hydroalcoholic extract concentrations (µg DPE/mL). Results are expressed as means ± standard deviations. Different letters indicate significant differences between samples (*p* < 0.05) according to Student’s *t* test.

Considering that the analyzed extracts are derived from byproducts of the wine industry and may harbor potential health benefits, it becomes crucial to assess their toxicity. This assessment employs two distinct experimental models: a holistic organism model utilizing *Artemia salina* and a eukaryotic cell model involving human red blood cells. Notably, at concentrations up to 1000 μg/mL, none of the extracts demonstrated toxicity towards red blood cells. In the brine shrimp test, the Malbec and Torrontés pomace extracts exhibited no toxicity up to 500 μg/mL (Figure 2). Aligning with these results, polar extracts (both aqueous and alcoholic) from red grape pomaces of Malbec and Syrah varieties in the Cuyo region of Argentina (Mendoza) did not induce toxicity in fish (*Danio rerio*) and crustaceans (*Artemia salina*) up to a concentration of 500 μg/mL. Similarly, no toxicity was observed in a murine macrophage cell line (RAW 264.7) up to a concentration of 1000 μg/mL [55]. In the case of hydroalcoholic extracts derived from white grape pomace (Falanghina) and red grape pomace (Tintilia and Vernaccia Nera di Serrapetrona) from Italy, the toxicity limits surpassed 40 mg/mL. In contrast, for the Sagrantino red variety, the toxic threshold was determined to be greater than 2 mg/mL in the brine shrimp test [56].

#### 3.2.4. Enzyme Inhibition

**Figure 3:**
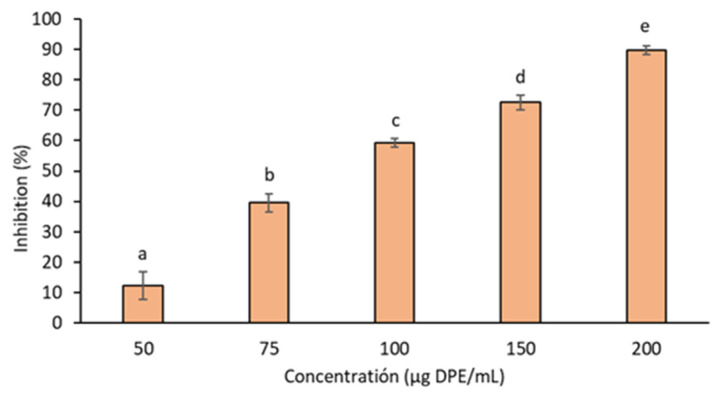
The tyrosinase inhibition present in the Malbec pomace hydroalcoholic extract. Results are expressed as means ± standard deviations. Different letters mean significant differences between concentrations (*p* < 0.05), according to Tukey’s test.

Skin aging often leads to pigmentation disorders, prompting the cosmetic industry to seek anti-hyperpigmentation compounds. These disorders occur due to melanin accumulation, which is influenced by various factors. Therefore, inhibiting melanin production by blocking the enzyme tyrosinase is a key strategy for skin-whitening products, making flavonoids promising compounds for this field [57]. Exploiting the bioactives contained in grape pomaces to obtain high-value cosmetics may support the growth of innovative start-ups and expand the value chain of grapes [9]. In this study, Torrontés demonstrated no impact on this enzyme activity at up to 200 µg/mL concentrations. Conversely, Malbec pomace displayed a dose-dependent inhibitory effect, leading to an approximately 90% decrease in tyrosinase activity at the same concentration (200 µg/mL) (Figure 3). The IC_50_ value for the Malbec extract was 89.9 ± 2.1 µg/mL. For kojic acid, a well-established tyrosinase inhibitor used as a reference, the IC_50_ was 0.37 ± 0.03 µg/mL. In comparison, the IC_50_ reported for Tempranillo (red variety) pomace extract (ethanol 50%) from Valladolid (Spain) was 4000 µg/mL [6]. Additionally, an ethanol extract (0.3 mL aliquots) of a mixture of white grape (Trebbiano and Verdicchio) pomaces demonstrated a 79% reduction in tyrosinase activity [58]. Extracts from the red grape stems of six grape varieties (1 mg/mL) inhibited the tyrosinase enzyme’s activity from 41.47% to 53.83%, with the Syrah variety exhibiting the highest activity [59]. Furthermore, it is noteworthy that certain flavonoids, including kaempferol, catechin, myricetin, and quercetin, among others, have been identified as inhibitors of tyrosinase in previous studies [6,58]. The higher content of flavonoid-type compounds in the Malbec extract could elucidate its superior ability to inhibit this enzyme. These results suggest that Malbec pomace from the Calchaquí Valleys is a valuable source of natural ingredients for cosmeceutical applications.

5-lipoxygenase (5-LOX) governs a critical biosynthetic pathway in generating eicosanoids. The ultimate product of the 5-LOX pathway, leukotriene B4 (LTB4), is a mediator in several diseases. Inhibitors of 5-LOX exhibit therapeutic potential for various inflammatory conditions, including asthma, allergies, and atherosclerosis [60]. The pivotal role of LOX and its derivatives in tumor initiation and cancer metastasis is well established. Heightened levels of 5-LOX have been discerned in diverse cancer cell lines, encompassing those linked to prostate, lung, colon, breast, and other cancers [61]. Thus, identifying compounds capable of inhibiting the LOX enzyme is a compelling focal point in the quest for bioactive natural products with potential anticancer properties.

**Figure 4:**
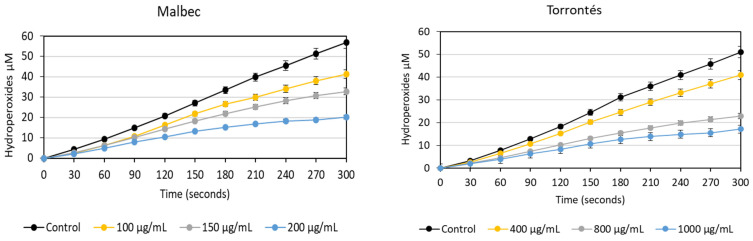
The lipoxygenase inhibition at different concentrations (µg DPE/mL) of hydroalcoholic extracts of Malbec and Torrontés pomaces. Results are expressed as means ± standard deviations.

Figure 4 illustrates the kinetics of hydroperoxide production by the LOX enzyme at varying concentrations of pomace extracts. Both compounds exhibit a dose-dependent inhibition of LOX activity. Based on the dose–response curves, the extract concentration resulting in a 50% activity reduction (IC_50_) was estimated to be 154.7 ± 4.1 and 784.7 ± 18.2 µg DPE/mL for Malbec and Torrontés, respectively, indicating that the Malbec extract is a more potent inhibitor of LOX than the Torrontés extract. The IC_50_ values for the positive control employed, namely quercetin and gallic acid, were 48.4 ± 1.9 and 54.1 ± 0.5 µg/mL, respectively. Grape pomace extracts from Montepulciano d’Abruzzo (Italy) red wine displayed dose-dependent LOX inhibitions ranging from 23% to 47% at tested concentrations of 10 and 100 µg/mL, respectively [62]. Previous studies have indicated a positive correlation between LOX inhibition and the concentrations of gallic acid, vanillic acid, p-coumaric acid, catechin, epicatechin, and rutin in pomaces [63]. In the present work, it was observed that the Malbec pomace exhibited higher levels of most of these compounds, potentially accounting for its fivefold greater capacity to inhibit this enzyme.

The xanthine oxidase inhibitors have potential as anti-gout agents. Hyperuricemia, a metabolic anomaly characterized by the excess production of uric acid or its insufficient excretion, often leads to gout, marked by elevated serum urate and the simultaneous accumulation of urate crystals in bones or joints. Xanthine oxidase converts hypoxanthine to xanthine and, subsequently, produces uric acid. Effective strategies for gout prevention and recovery include xanthine oxidase inhibitors. Grape polyphenols, including cinnamic acid derivatives, syringic acid, ellagic acid, caffeic acid, and ferulic acid, are considered to contribute to the inhibition of xanthine oxidase activity, as mentioned in a review of several in vitro studies [64]. Despite the potential of phenolic compounds and flavonoids in mitigating the conditions associated with hyperuricemia, in the trials performed, none of the pomace extracts were able to inhibit the enzyme up to a concentration of 250 µg/mL. This study marks the first report on the evaluation of wine pomaces from the Calchaquí Valleys concerning LOX, tyrosinase, and xanthine oxidase enzymes.

## 4. Conclusions

In this study, we identified 35 phenolic compounds in the hydroalcoholic extracts derived from the pomace of Malbec and Torrontés wines from the Calchaquí Valleys of Argentina. Both extracts exhibited significant antioxidant activity, with Malbec showing superior antiradical activity, reducing power, hypochlorite scavenging, and iron-chelating capacity. Additionally, Malbec extract demonstrated the ability to inhibit tyrosinase, an enzyme affecting the skin, and the pro-inflammatory enzyme lipoxygenase. However, the Torrontés pomace extract was more effective against colon cancer cells.

These findings support the idea that both extracts could be considered potential functional ingredients with preventive properties against diseases. These extracts have potential applications in pharmacology, cosmetics, and the food industry. Therefore, it is imperative to conduct further comprehensive studies to validate their practical utilization. The exploitation of this byproduct has the potential to impact local economies significantly.

## Figures and Tables

**Table 1:** The polyphenol composition of the extracts.

Phytochemical Group	Malbec	Torrontés
	mg/g DPE	mg/g DPE
Total Phenolics (GAE)	156.01 ± 3.49 ^a^	19.91 ± 1.21 ^b^
Hydroxycinnamic acids (CAE)	11.39 ± 0.32 ^a^	0.47 ± 0.01 ^b^
Orthodiphenols (CAE)	31.79 ± 0.62 ^a^	4.71 ± 0.28 ^b^
Anthocyanins (C3GE)	6.30 ± 0.49 ^a^	<LOQ
Non-flavonoid phenolics (GAE)	30.49 ± 1.15 ^a^	8.01 ± 0.94 ^b^
Tannins (CE)	23.20 ± 1.30 ^a^	17.37 ± 0.47 ^b^
Total flavonoids (QE)	327.25 ± 6.30 ^a^	43.67 ± 1.39 ^b^
Flavones/Flavonols (QE)	73.56 ± 1.41 ^a^	1.17 ± 0.07 ^b^
Flavanones/Dihydroflavonols (NE)	63.65 ± 5.56 ^a^	10.10 ± 0.85 ^b^

DPE: dry pomace extract. DP: dry pomace. GAE: gallic acid equivalents. CAE: caffeic acid equivalents. QE: quercetin equivalents. NE: naringenin equivalents. CE: catechin equivalents. C3GE: cyanidin-3-glucoside equivalents. LOQ: limit of quantification. Results are expressed as means ± standard deviations. Different letters in the same row indicate significant differences between samples (*p* < 0.05) according to Student’s *t* test.

**Table 2:** The phenolic compounds quantified in the wine pomace samples through HPLC-DAD.

Phenolic Compound	Retention Time	mg/100 g DPE		mg/100 g DP	
	(min)	Malbec	Torrontés	Malbec	Torrontés
Phenolic acids					
Gallic acid	5.618	245.0 ± 12.0 ^a^	89.3 ± 4.5 ^b^	39.2 ± 1.9 ^a^	65.2 ± 3.3 ^b^
Protocatechuic acid	9.935	700.0 ± 35.0 ^a^	71.2 ± 3.6 ^b^	112.0 ± 5.6 ^a^	51.9 ± 2.6 ^b^
Neochlorogenic acid	10.219	6.1 ± 0.3 ^a^	1.9 ± 0.1 ^b^	0.9 ± 0.1 ^a^	1.4 ± 0.1 ^b^
Caftaric acid	15.436	<LOQ	90.6 ± 4.5	<LOQ	66.1 ± 3.3 ^b^
Chlorogenic acid	17.869	6.6 ± 0.3 ^a^	6.8 ± 0.3 ^a^	1.1 ± 0.1 ^a^	5.0 ± 0.2 ^b^
4-*O*-caffeoylquinic acid	19.897	130.0 ± 6.0 ^a^	118.0 ± 6.0 ^a^	20.8 ± 1.0 ^a^	86.1 ± 4.4 ^b^
Vanillic acid	20.748	ND	ND	ND	ND
Caffeic acid	21.224	6.7 ± 0.31 ^a^	18.7 ± 0.9 ^b^	0.9 ± 0.1 ^a^	13.7 ± 0.7 ^b^
Syringic acid	22.283	38.6 ± 1.9 ^a^	27.6 ± 1.4 ^b^	6.2 ± 0.3 ^a^	20.2 ± 1.0 ^b^
*p*-Coumaric acid	33.758	28.2 ± 1.4 ^a^	36.7 ± 1.8 ^b^	4.5 ± 0.2 ^a^	26.8 ± 1.3 ^b^
*trans*-Ferulic acid	37.289	45.7 ± 2.3 ^a^	74.2 ± 3.7 ^b^	7.3 ± 0.4 ^a^	54.2 ± 2.7 ^b^
Sinapic acid	37.662	108.0 ± 5.0 ^a^	89.6 ± 4.5 ^b^	17.3 ± 0.8 ^a^	65.4 ± 3.3 ^b^
3,5-di-*O*-caffeoylquinic acid	50.127	7.2 ± 0.4 ^a^	45.0 ± 2.2 ^b^	1.2 ± 0.1 ^a^	32.9 ± 1.6 ^b^
Ellagic acid	55.284	33.4 ± 1.7	ND	5.3 ± 0.3	ND
4,5-di-*O*-caffeoylquinic acid	56.781	50.7 ± 2.5 ^a^	258.0 ± 13.0 ^b^	8.1 ± 0.4 ^a^	188.3 ± 9.5 ^b^
Cinnamic acid	58.47	ND	ND	ND	ND
Flavonoids					
(+)-Catechin	14.143	618.0 ± 31.0 ^a^	70.1 ± 3.5 ^b^	98.9 ± 5.0 ^a^	51.2 ± 2.6 ^b^
(−)-Epicatechin	23.294	43.2 ± 2.2 ^a^	17.2 ± 0.9 ^b^	6.9 ± 0.4 ^a^	12.6 ± 0.7 ^b^
Naringin	49.847	31.9 ± 1.6 ^a^	54.6 ± 2.7 ^b^	5.1 ± 0.3 ^a^	39.9 ± 2.0 ^b^
Quercetin-3-*O*-galactoside	52.177	25.2 ± 1.3 ^a^	43.8 ± 2.2 ^b^	4.0 ± 0.2 ^a^	32.0 ± 1.6 ^b^
Quercetin-3-*O*-glucopyranoside	52.735	ND	ND	ND	ND
Rutin	53.284	19.1 ± 1.0 ^a^	16.4 ± 0.8 ^b^	3.1 ± 0.2 ^a^	12.0 ± 0.6 ^b^
Phloridzin	54.355	32.3 ± 1.6 ^a^	ND	5.2 ± 0.3	ND
Myricetin	57.943	17.6 ± 0.9 ^a^	18.9 ± 0.9 ^a^	2.8 ± 0.1 ^a^	13.8 ± 0.7 ^b^
Quercitrin	59.07	ND	ND	ND	ND
Kaempferol-3-*O*-glucoside	59.466	ND	116.0 ± 6.0	ND	84.7 ± 4.4
Kaempferol-3-*O*-rutinoside	60.01	<LOD	66.6 ± 3.3	<LOD	48.6 ± 2.4
Isorhamnetin-3-*O*-glucoside	60.277	ND	95.6 ± 4.8	ND	69.8 ± 3.5
Isorhamnetin-3-*O*-rutinoside	61.568	24.2 ± 1.2 ^a^	10.2 ± 0.5 ^b^	3.9 ± 0.2 ^a^	7.4 ± 0.4 ^b^
Naringenin	68.149	<LOD	<LOD	<LOD	<LOD
Quercetin	71.031	18.6 ± 0.9 ^a^	11.4 ± 0.6 ^b^	3.0 ± 0.1 ^a^	8.3 ± 0.4 ^b^
Phloretin	72.269	<LOQ	<LOD	<LOQ	<LOD
Tiliroside	76.233	22.7 ± 1.1	<LOQ	3.6 ± 0.2	<LOQ
Kaempferol	79.854	3.4 ± 0.2 ^a^	4.4 ± 0.2 ^b^	0.6 ± 0.1 ^a^	3.2 ± 0.2 ^b^
Apigenin	81.44	<LOD	<LOD	<LOD	<LOD
Chrysin	90.832	<LOD	<LOD	<LOD	<LOD
Stilbenoids and others					
*trans*-Polydatin	39.182	18.9 ± 0.9 ^a^	3.6 ± 0.2 ^b^	3.0 ± 0.1 ^a^	2.6 ± 0.1 ^b^
Resveratrol	52.507	ND	ND	ND	ND
*trans*-Epsilon viniferin	69.158	6.5 ± 0.3 ^a^	6.2 ± 0.3 ^a^	1.0 ± 0.1 ^a^	4.5 ± 0.2 ^b^

DPE: dry pomace extract. DP: dry pomace. Results are expressed as means ± standard deviations. LOQ: limit of quantification; LOD: limit of detection; ND: not detected. Different letters in the same row indicate significant differences between samples (*p* < 0.05) according to Student’s *t* test.

**Table 3:** The antioxidant capacities of the wine pomace hydroalcoholic extracts.

Sample	Phosphomolybdenum Reducing Capacity (μgAAE/mg DPE)	Cupric Reducing Capacity (μgGAE/mg DPE)	ABTS˙+ Scavenging IC_50_ (μg/mL)	NO Scavenging IC_50_ (μg/mL)	Fe^3+^ Reducing RC_50_ (μg/mL)	Iron Chelating CC_50_ (μg/mL)	O_2_^●−^ Scavenging IC_50_ (µg/mL)	HOCl Scavenging IC_50_ (µg/mL)
Malbec	178.57 ± 4.99 ^a^	171.18 ± 2.2 ^a^	7.79 ± 0.17 ^a^	414.19 ± 5.79 ^b^	10.22 ± 0.16 ^a^	41.82% ± 0.48% *	74.17 ± 4.12 ^a,b^	6.71 ± 0.36 ^b^
Torrontés	4.74 ± 0.16 ^b^	26.12 ± 0.52 ^b^	49.5 ± 1.46 ^b^	15.34% ± 0.78% **	84.62 ± 0.95 ^b^	11.28% ± 1.05% *	874.61 ± 15.71 ^c^	27.40 ± 0.19 ^c^
Controls								
BHT	-	-	-	-	11.37 ± 0.13 ^a^	-	-	-
Trolox	-	-	3.74 ± 0.06 ^a^	-	-	-	-	-
Ascorbic acid	-	-	-	36.13 ± 6.01 ^a^	-	-	-	-
EDTA	-	-	-	-	-	13.97 ± 0.06	-	-
Catechin	-	-	-	-	-	-	99.21 ± 0.85 ^b^	0.095 ± 0.006 ^a^
Gallic acid	-	-	-	-	-	-	52.49 ± 1.58 ^a^	0.82 ± 0.06 ^a^

Different letters in the same column show significant differences among each treated group, according to a Tukey test (*p* ≤ 0.05). IC_50_ = the concentration required to demonstrate a decrease of 50% in the reactivity of the reactive species in the tested media (mean ± standard error of the mean). The phosphomolybdenum-reducing capacity is expressed as micrograms of ascorbic acid equivalents per milligram of DPE (dry pomace extract) (µg AAE/mg DPE). The cupric-reducing antioxidant capacity is expressed as micrograms of gallic acid equivalents (μg GAE/mg DPE). The Fe^3+^-reducing capacity (RC), ABTS radical cation (ABTS˙+)-, nitric oxide (NO)-, superoxide anion radical (O_2_^●−^)-, and hypochlorous acid (HOCl)-scavenging capacities (IC). The iron-chelating capacity (CC) determined through linear regression analysis. * An inhibition percentage with the concentration of 1000 µg/mL. ** An inhibition percentage with the concentration of 250 µg/mL.

**Table 4:** The effects of the wine pomace samples’ exposure on the viability of HT29-MTX and Caco-2 cells at different concentrations as measured via an MTT assay (*n* = 3).

	Cell Viability (%)			
Concentration (µg DPE/mL)	HT29-MTX Cells		Caco-2 Cells	
	Malbec	Torrontés	Malbec	Torrontés
0.1	132.25 ± 10.18 ^a^	109.33 ± 19.90 ^a^	120.67 ± 6.92 ^a^	70.29 ± 10.03 ^b^
1.0	133.03 ± 12.93 ^a^	110.34 ± 9.63 ^a^	79.08 ± 6.94 ^b^	65.14 ± 14.27 ^b^
10	131.85 ± 14.80 ^a^	103.57 ± 16.69 ^a^	82.82 ± 11.80 ^b^	66.85 ± 7.36 ^b^
100	122.19 ± 16.94 ^a^	66.87 ± 13.06 ^b^	88.48 ± 12.45 ^b^	66.85 ± 10.52 ^b^
1000	104.75 ± 23.03 ^b^	33.56 ± 4.38 ^c^	81.26 ± 12.83 ^b^	52.51 ± 1.53 ^c^
Medium	100.00 ± 8.86 ^a^			
Triton X-100	0.00 ± 0.00			

Different letters mean significant differences between concentrations of the same extract (*p* < 0.05), according to Tukey’s test.

## Data Availability

The data presented in this study are available on request from the corresponding author. The data are not publicly available due to privacy restrictions.
